# The Free-Amino-Acid Content in Six Potatoes Cultivars through Storage

**DOI:** 10.3390/molecules26051322

**Published:** 2021-03-02

**Authors:** Anna Pęksa, Joanna Miedzianka, Agnieszka Nemś, Elżbieta Rytel

**Affiliations:** Department of Food Storage and Technology, Wroclaw University of Environmental and Life Sciences, Chelmonskiego 37 Street, 51-630 Wroclaw, Poland; anna.peksa@upwr.edu.pl (A.P.); agnieszka.nems@upwr.edu.pl (A.N.); elzbieta.rytel@upwr.edu.pl (E.R.)

**Keywords:** coloured potatoes, time of storage, temperature of storage, free amino acids pool

## Abstract

Potatoes of six cultivars (*Solanum tuberosum* L.) with red, purple, and yellow flesh were stored at 2 and 5 °C for 3 and 6 months, and the influence of these factors on the content of free amino acids was determined. The potato cultivar and storage time had the greatest impact on the free amino acid content. The tubers of red-fleshed (Rote Emma) and purple-fleshed (Blue Congo) potatoes contained over 28 mg/g DM of free amino acids, and the Blaue Annelise cultivar with purple flesh had over 18 mg/g DM. After 6 months, the highest increase in their content (by 36%) was recorded in tubers of the Fresco cultivar (yellow-fleshed). In the analysed potatoes, the content of alanine, proline, serine, γ-aminobutyric acid, and α-aminoadipic acid increased, while that of asparagine, aspartic acid, and glutamine decreased. Asparagine decreased to the greatest extent in “Blaue Annelise” potatoes (by 24%) and that of glutamine in tubers of Rote Emma and Vineta by 18%.

## 1. Introduction

Potatoes are an excellent source of nutrients and energy and provide only trace amounts of fat and salt [1], giving them a high nutritional value. The anthocyanins found in potatoes with purple and red flesh enrich the pool of tuber polyphenols in amounts depending on the cultivar, flesh colour, and environmental conditions compared with cultivars with traditional white-, cream-, or yellow-flesh. Thus, they significantly increase the antioxidant potential of potatoes and their health-promoting effects. For example, they have anti-inflammatory, antidiabetic and cancer-inhibiting effects [2,3,4,5].

According to studies by various authors [6,7,8,9,10], the share of protein nitrogen of total nitrogen in potatoes ranges from 37% to over 63%, i.e., in the range from 0.24% to 0.36% of fresh weight. Approximately 49% of the total amino acids are in free form [11], and the largest share, reaching 34–90% of the total pool of free amino acids, are the asparagine and glutamine amides [12,13]. Some authors [12,14] analysed purple- and red-fleshed potatoes for amino acid content and tuber nutritional value. The content of nonprotein amino acids and amino acid metabolites in potatoes of various cultivars, including those with purple flesh, were also studied and analysed [15]. The presence of such compounds as β-alanine (β-Ala), α-aminoadipic acid (α-AAA), α-aminobutyric acid (α-ABA), γ-aminobutyric acid (GABA), hydroxylysine (Hyl), L- carnitine (L-Car), ethylamine (EtNH2), or L-ornithine (Orn) was found. Choi et al. [15] and Ito et al. [16] emphasized that the nutritional role of these free forms of amino acids is largely unknown despite the fact that there are many reports of their biological activity as components of cell membranes or substances acting on specific receptors in animal and human bodies. They also noted that these compounds have been known as components of coenzymes, substances that regulate or affect water management and the correct concentration of electrolytes in living organisms, supporting the removal of ammonia from the blood or the action of neurotransmitters in the central nervous system [15,16].

Long-term storage, usually at 4 °C or higher (6–10 °C), may cause large fluctuations in the dry-matter content of potato tubers, which are more concerned with carbohydrates, proteins, and amino acids [17]. Cultivars with coloured flesh, especially purple, are suitable for long-term storage [5]. Therefore, they can be not only a valuable food product but also a raw material for the production of various products, including fried snacks [18]. Despite many studies conducted with increased intensity for over 15 years in various research centres around the world, potatoes with coloured flesh are still new to the food market.

According to various authors, the storage conditions and cultivar characteristics of potatoes have a large impact on the content of nitrogen compounds and their structure [12,19,20]. This is related to the diversified activity of proteolytic enzymes in the breakdown of proteins, depending on the cultivar and storage conditions. The changes that occur are important for both the consumer and potato processors, because the additionally formed free amino acids, such as glutamic and aspartic acid, serine, valine, proline, and glutamine, play an important role in shaping the taste of tubers. Changes in the composition of nitrogen compounds may also affect the quality characteristics of food products obtained from them [6]. The studies of changes in the content and profile of free amino acids in potatoes, conducted by various authors, referred to the influence of vegetation and environmental conditions, such as “water stress” [11,21], temperature, and storage time of tubers [6,22], but did not apply to cultivars with coloured flesh.

It is believed that free amino acids are an important indicator of the technological quality of tubers due to their participation in Maillard browning and have an ambiguous effect on the disturbance of the relationship between the sugar content and colour of crisps. According to some authors [22], the selection of potato cultivars used for the production of fried products should primarily consider the concentration of reducing sugars and the content of free asparagine and other free amino acids. Potatoes with purple or red flesh are still not well researched in terms of the content of free amino acids in tubers, which is the result of long-term storage at low temperatures.

In related studies [23], it was shown that the storage of potato cultivars with red, purple, and yellow flesh at low temperatures, i.e., 2 and 5 °C, resulted in a decrease in the content of amino acids depending on the storage time and potato cultivar. These studies concerned the amino acids determined after earlier hydrolysis of the samples and did not reflect the content of free forms of amino acids, especially that acid hydrolysis of proteins leads to the loss of the content of such amino acids as glutamine, asparagine, and tryptophan. These amino acids are not measurable due to their transformation into the appropriate acids. In addition, there is observed a loss of other amino acids, such as, for example, the metabolites GABA and ornithine [13,24]. Changes in the content and structure of free forms of amino acids during the necessary long-term storage of potato tubers at low temperatures may be of significant informative importance. Moreover, it is the fact that the content of these compounds in purple- and red-fleshed potatoes, rich in anthocyanins, increases the health-promoting effect of this food product popular among consumers around the world. Therefore, due to the growing popularity of potatoes of varieties with coloured flesh among consumers and producers of potato products, the aim of the research was to determine the content of free amino acids in potatoes of selected cultivars with red and purple flesh, considered as promising in terms of their sensory and technological properties, during long-term storage at low temperatures, compared to cultivars with traditional yellow flesh, popular in industry and among consumers.

## 2. Results

### 2.1. Effect of the Applied Research Factors

Based on the conducted studies, it was revealed that the content of the sum of free amino acids and most of the free amino acids determined in the tested potatoes of six cultivars, apart from isoleucine and ethylamine, largely depended on the cultivar and its interaction with tuber storage conditions, i.e., time and temperature. This was demonstrated by the statistical analysis of data in the ANOVA test for three variables (Table 1), both at the significance level *p* < 0.05, *p* < 0.01, and *p* < 0.001. The storage period of tubers influenced to a large extent (*p* < 0.01 and *p* < 0.001) the content of most of the determined free amino acids and to a lesser extent (*p* < 0.05) the content of glutamic acid, arginine, AAA metabolites, and ornithine. On the other hand, the temperature had a smaller influence on the content of free amino acids in potatoes during long-term storage. It mainly influenced the content of asparagine, aspartic acid, glutamine, arginine, proline, alanine, serine, and the content of lysine and histidine, and to the least extent (*p* < 0.05) on the content of glutamic acid, threonine, phenylalanine, glycine, and the AAA metabolite.

Due to the predominant influence of potato cultivar and its interaction with tuber storage time and temperature on the content of free amino acids, the impact of these factors was discussed in detail later in this article.

### 2.2. Effect of the Potato Cultivar

Potatoes of purple-, red-, and yellow-fleshed cultivars used in the study differed significantly in terms of the amount of free amino acids in their tubers. It resulted from both the varietal differences and the different cultivation places of cultivars with coloured flesh (Přerov nad Labem in Czech Republic) and cultivars with a traditional yellow colour (Lower Silesia in Poland). They contained on average from 18.53 to 28.55 mg/g DM of all free amino acids, among which the largest share was asparagine (Asn) and glutamine (Gln) (Table 2). The share of asparagine in the total free amino acids ranged from 28.1%, in potatoes of the Rote Emma cultivar with red flesh, to 38.9% in the tubers of Vineta cultivar with yellow flesh, and the share of glutamine (Gln) ranged from 11.5%, in “Blaue Annelise” purple potatoes, to 23.9% in the tubers of Rote Emma cultivar (Table 2). In the studies presented by Brierley et al. [12], the amides of asparagine and glutamine accounted together from 50% to 90% of the total free amino acids; therein, free asparagine usually accounted for one third of the total pool of free amino acids. Large variation in the content of total free amino acids between cultivars, including free asparagine and glutamine, were also confirmed by other authors [22,25]. The highest amounts of asparagine (9.79–9.31 mg/g DM) were found in tubers of red-fleshed Herbie 26 and yellow-fleshed Fresco and the lowest in purple-fleshed “Blaue Annelise” potatoes (5.49 mg/g DM). While the highest glutamine content was found in potatoes of the Rote Emma cultivar (6.78 mg/g DM), much smaller amounts of this amide were found, for example, in the tubers of Blaue Annelise and Vineta cultivars, 2.12 and 2.40 mg/g DM, respectively. Regardless of the storage conditions, the cultivars with the highest total content of free amino acids were red-fleshed Rote Emma and purple-fleshed Blue Congo, and the least of these compounds was determined in purple-fleshed Blaue Annelise tubers and yellow-fleshed Vineta.

The differentiation in the content of free amino acids in the analysed tubers of six cultivars was probably related to the variable activities of proteolytic enzymes present in them. They play an important role in regulating the levels of free amino acids in potato tubers during storage [12]. The proportionally high content of free amino acids in potatoes can increase their nutritional value. For example, essential amino acids such as valine, leucine, and isoleucine, called dietary branched amino acids, are absorbed much faster than single (unbranched) amino acids and can be involved in building muscle and increasing the strength of the human body [16]. Among the analysed tubers, the highest amounts of these free amino acids were found in potatoes of the Blue Congo cultivar (Table 2). According to the research of Sato et al. [26], potatoes characterised by an increased content of free amino acids, especially glutamine, lysine, arginine, valine, phenylalanine, histidine, asparagine, glutamic and aspartic acid, and tyrosine are considered egumi-flavored products (spicy, slightly bitter, and tart taste). Among the studied potatoes, tubers of the Rote Emma, Blue Congo, and Fresco cultivars can be especially considered as products with such a taste, as can tubers of the Rote Emma cultivar due to their high content of glutamine; Blue Congo and Fresco cultivars due to their high content of glutamic acid, tyrosine, isoleucine, and histidine; and Blue Congo due to their high content of arginine. Certain free amino acids, including glycine, alanine, and proline, but also serine, give the product a sweet taste. Potatoes of the Fresco cultivar contained not only more amino acids that produce an egumi taste but also more proline, serine, and glycine, compared with potatoes of other cultivars. This may have contributed to the characteristic complex flavour of these potatoes [16].

The differentiation of the analysed potato samples of six cultivars in terms of the content of free amino acids ranged from two to over five times, regardless of flesh colour. The content of free amino acids such as threonine, α-aminoadipic acid (α-AAA), arginine, serine, glutamine, lysine, phenylalanine, and γ-aminobutyric acid (GABA) was highly variable. In all analysed potatoes, a very low content of glycine, cystine, and ornithine was found, below 0.1 mg/g DW (0.03–0.09) (Table 2), which was also confirmed by other authors [16].

γ-Aminobutyric acid (GABA) is an important metabolite present in food, including potatoes, due to its positive effect on human health. Its content in the analysed potatoes varied within wide limits, from 0.57 (Vineta) to 1.66 mg (Fresco) in 1 g of tuber dry weight. Based on 150 g of potatoes consumed at one time, the tested samples, stored from 3 to 6 months, would provide 32–63 mg of GABA. According to Choi et al. [15], potatoes may show large differences in terms of the content of this amino acid, depending on the cultivar but also on the part of the tuber. GABA is an important ingredient in fermented foods popular in Japan. It is believed that its daily supplementation in the amount of 10–20 mg reduces blood pressure in humans [27].

### 2.3. Effect of Storage Temperature and Cultivar

**Figure 1:**
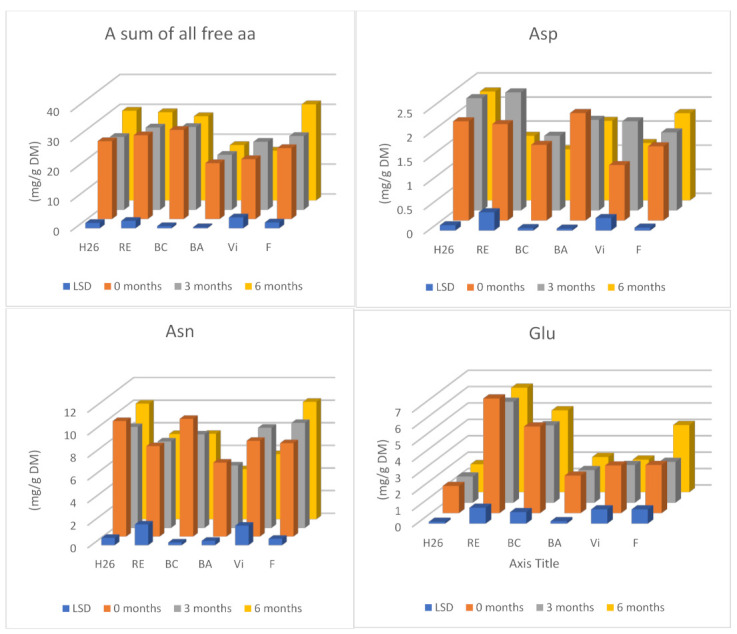

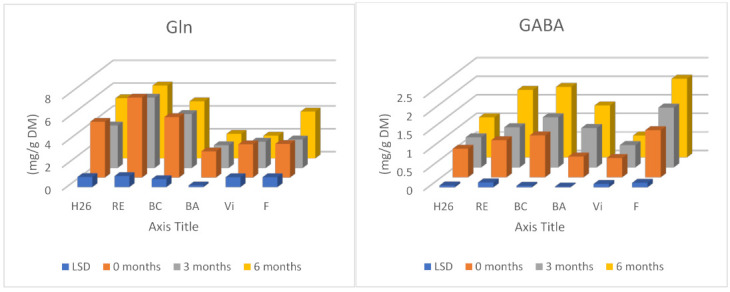
The effect of storage time on the content of chosen free amino acids (Asp, Asn, Glu, Gln, and GABA) and sum of all free amino acids in potatoes of six cultivars (average results of storage temperature). Each data value is presented as the mean ± standard deviation (*n* = 4); H26—Herbie 26, RE—Rote Emma, BC—Blue Congo, BA—Blaue Annelise, V—Vineta, F—Fresco; Asn—asparagine, Asp—aspartic acid, Gln—glutamine, Glu—glutamic acid, and GABA-γ—aminobutyric acid.

Regardless of flesh colour, the analysed potatoes contained, on average, 8% more total free amino acids after storage at 2 °C than at 5 °C (Table 3). Tubers stored at a lower temperature contained, in particular, more proline (approximately 24%), glutamine (approximately 14%), aspartic acid (approximately 12%), and serine (approximately 11%), as well as more asparagine, glutamic acid, arginine, histidine, and phenylalanine, in the range of 7–9%. A significant influence of the varietal factor was noted, especially on the content of aspartic and glutamic acids, asparagine, glutamine, and GABA (Figure 1). In contrast to other tubers, potatoes of the Vineta cultivar contained less asparagine and aspartic acid after storage at 2 °C than at 5 °C. Matsuura-Endo et al. [17], when analysing the chemical composition of potatoes useful for the production of french fries, found that they contained large amounts of asparagine during long-term storage of tubers at low temperatures (2–6 °C). These authors also confirmed the significant influence of the potato cultivar on the accumulation of aspartic and glutamic acids under these conditions.

The storage temperature had a significant effect on the content of GABA, but this depended on the potato cultivar. Tubers of the Herbie 26 and Blaue Annelise cultivars stored at 2 °C contained more of this form of free amino acid than those stored at 5 °C. The content of the sum of free amino acids and glutamine in potatoes of the Fresco cultivar did not depend on the tuber storage temperature. In previous studies [12,13], the storage temperature of tubers with a traditional flesh colour had a smaller effect on the content of total free amino acids in the first period not longer than 6 months. According to various authors [12,13,17], low temperatures (2–6 °C) can increase the content of free amides (asparagine and glutamine) in potatoes during long-term storage. This increase is also closely related to the increase in the sum of free amino acids, of which these amides constitute the majority. According to Brierley et al. [12], the increase in the content of free amino acids in potatoes during storage is, to a greater extent, the effect of the enzyme glutamine synthetase and the amidation of amino acids released from patatin proteins and, to a lesser extent, also the effect of protein hydrolysis and deamidation. In the abovementioned studies, some cultivars, such as Pentland Dell, accumulated larger amounts of free amino acids, including amides, especially during storage at 10 °C.

### 2.4. Effect of Storage Time and Cultivar

**Figure 2:**
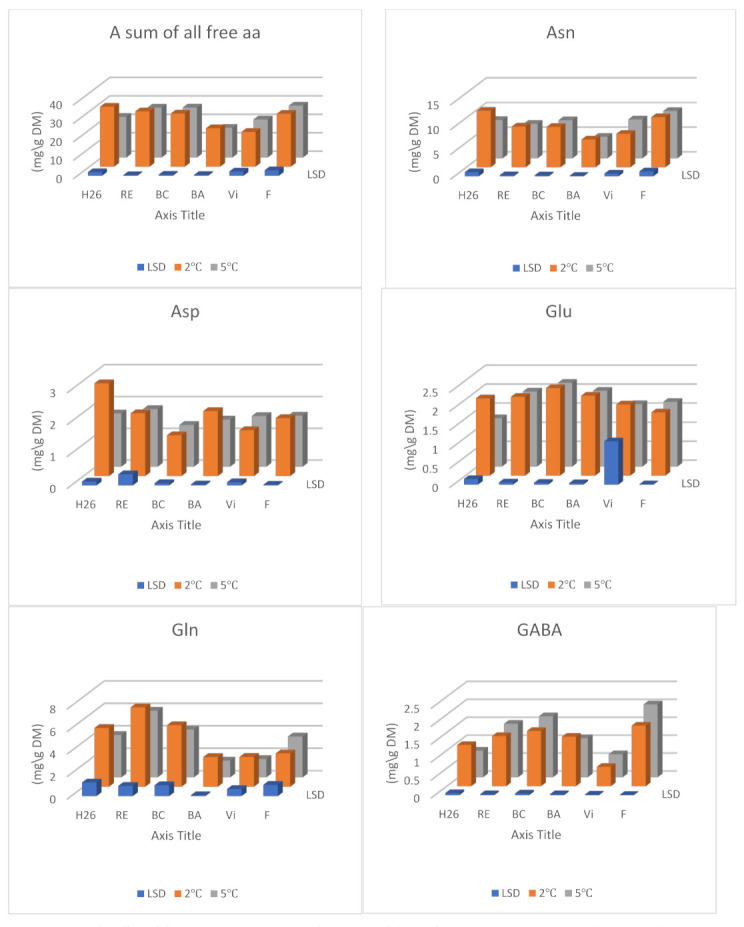
The effect of the storage temperature on the content of chosen free amino acids (Asn, Asp, Glu, Gln, and GABA) and sum of all free amino acids in potatoes of six cultivars (average results of the time of storage). Each data value is presented as the mean ± standard deviation (*n* = 6); H26—Herbie 26, RE—Rote Emma, BC—Blue Congo, BA—Blaue Annelise, V—Vineta, and F—Fresco; and Asn—asparagine, Asp—aspartic acid, Gln—glutamine, Glu—glutamic acid, and GABA-γ—aminobutyric acid.

Only extending the storage time of tubers at low temperatures from 3 to 6 months resulted in significant changes in their free amino acid content. After this time, the analysed potatoes contained, on average, approximately 6% more free amino acids (Table 4) compared with tubers before storage. However, a significant effect of the potato cultivar on the content of total free amino acids in tubers during storage was noted. In potatoes of the Herbie 26 and Fresco cultivars, there was an increasing trend; while in tubers of the Blue Congo and Vineta cultivars, there was a decreasing trend (Figure 2). There was a significant increase in the content of the sum of free amino acids after 6 months in potatoes of the yellow-fleshed Fresco cultivar (by an average of 36%) and in red-fleshed Herbie 26 (by an average of 15%). After 6 months of storage, the greatest amounts of free amino acids (30–32 mg/g DM) were found in the tubers of the yellow-fleshed Fresco and red-fleshed Herbie 26 and Rote Emma cultivars.

Other authors [12,13,22], while analysing various potato cultivars with the traditional light-fleshed colour, found a significant increase in the content of total free amino acids after 3–4 months of storage. After 6 months, those potatoes differed in the direction and size of changes in the content of these compounds. In most of the potatoes of the 10 cultivars studied by Halford et al. [22], the content of total free amino acids clearly decreased; while in tubers of the Pentland Dell cultivar, total free amino acids increased by an average of 65%. The differences in total free amino acid content between the cultivars studied by these authors reached 500% after 8 months of storage.

After 6 months of storage, the content of such amino acids as alanine (by approximately 70%), proline (by approximately 59%), serine (by approximately 48%), GABA and glycine (by 42–44%), and the metabolite α-AAA were especially increased (by approximately 39%). The content of tyrosine, histidine, and lysine also increased by 22–26% (Table 4). Other authors [16] confirmed the effect of long-term storage on the intensive accumulation, especially of proline, arginine, methionine, glutamic acid, and GABA, considering storage as a stress factor causing an increase in the content of some free forms of amino acids in potatoes. In the conducted studies, only the content of such amino acids as asparagine, aspartic acid, methionine, and L-ornithine were significantly reduced by an average of 15–33%. These studies also showed a decrease in the share of asparagine and glutamine in total free amino acids, especially after longer-term storage of tubers. The share of asparagine decreased to the greatest extent in potatoes of purple-fleshed “Blaue Annelise” (by approximately 24%), and the share of glutamine in tubers of the Rote Emma and Vineta cultivars (by approximately 18%). This could prove the diversified activity of potatoes of different cultivars in the mobilization of nitrogen reserves in tubers, aimed at the formation of sprouts [12,22,28,29].

Due to the high proportion of free amino acids in the total amino acid content, the impact of the potato cultivar on the content of such amino acids as asparagine, glutamine, aspartic and glutamic acids, and GABA in tubers during storage was determined. In all cultivars, except tubers of the Fresco cultivar, a decrease in asparagine was shown as a result of storage. Its content increased only in Fresco tubers, after both 3 (by an average of 12%) and 6 months (by an average of 26%) and decreased especially in potatoes of the Blue Congo and Blaue Annelise cultivars by 27% and 32%, respectively, after 6 months of storage. The changes in glutamine content depended on both the storage time and the cultivar. In potatoes of the Herbie 26, Rote Emma, Blaue Annelise, and Vineta cultivars, there was noted a decrease in the content of glutamine, mainly in tubers of the Herbie 26 cultivar (by an average of 24%) after 3 months and in the Vineta cultivar (by an average of 32%) after 6 months of storage. The glutamine content changed in the opposite direction in “Fresco” potatoes, which, after 6 months, contained on average 39% more of this amino acid. In studies by Brierley et al. [13], the greatest changes in the total content of asparagine and glutamine in potatoes concerned the Pentland Dell cultivar. The content of amides in tubers of this cultivar increased by 3–4 times after 25 weeks of storage, especially at higher temperatures (10 °C).

Slight changes were found in the content of glutamic acid, reaching 12–13%, in the period of 3–6 months of storage, which concerned only three potatoes, i.e., “Blue Congo”, “Fresco”, and “Blaue Annelise”. In potatoes of the Blue Congo and Blaue Annelise cultivars, they had a decreasing character and in tubers of the Fresco cultivar, an increasing one. Relatively stable amounts of glutamic acid in stored potatoes, regardless of flesh colour, were beneficial due to the participation of free forms of this amino acid in increasing nutrient absorption [16]. Despite the general tendency for aspartic acid to decrease in potatoes during long-term storage, an increase in the content of this amino acid after 3 months was shown in tubers of the Herbie 26 and Vineta cultivars, by 13% and 62%, respectively, and in Fresco tubers, after 6 months. The content of aspartic acid decreased to the greatest extent in potatoes stored for 6 months, in Blaue Annelise, Blue Congo, and Rote Emma cultivars (by 26–36%).

The content of the metabolite GABA clearly increased in most analysed potatoes, after both 3 and 6 months of storage, except in the Vineta cultivar. The content of this amino acid increased to the greatest extent in potatoes of the Blaue Annelise and Rote Emma cultivars after 6 months, which contained, respectively, 150% and 107% more GABA than unstored tubers. The increase in the content of GABA with a simultaneous slight decrease in the content of glutamic acid, especially in potatoes of the Blue Congo and Blaue Annelise cultivars, could have resulted from the use of glutamic acid for the synthesis of the metabolite GABA [27]. After 6 months of storage, the analysed potatoes contained an average of 1.48 mg/g DW of GABA, and the highest amounts were found in yellow-fleshed Fresco tubers (2.12 mg/g DW) and in purple-fleshed Blue Congo potatoes (1.90 mg/g DW) (Figure 2). The increase in the GABA content of potatoes with the extension of their storage period can be considered beneficial due to its positive effect on human health [16,30,31]. Studies have shown that potatoes, including stored potatoes, can be a good source of GABA, like some other vegetables. The content of GABA acid in vegetables varies widely. Good sources of GABA include pepper (3.73 mg/100 g), okra (7.59 mg/100 g), and onion (0.51 mg/100 g) [16].

**Figure 3:**
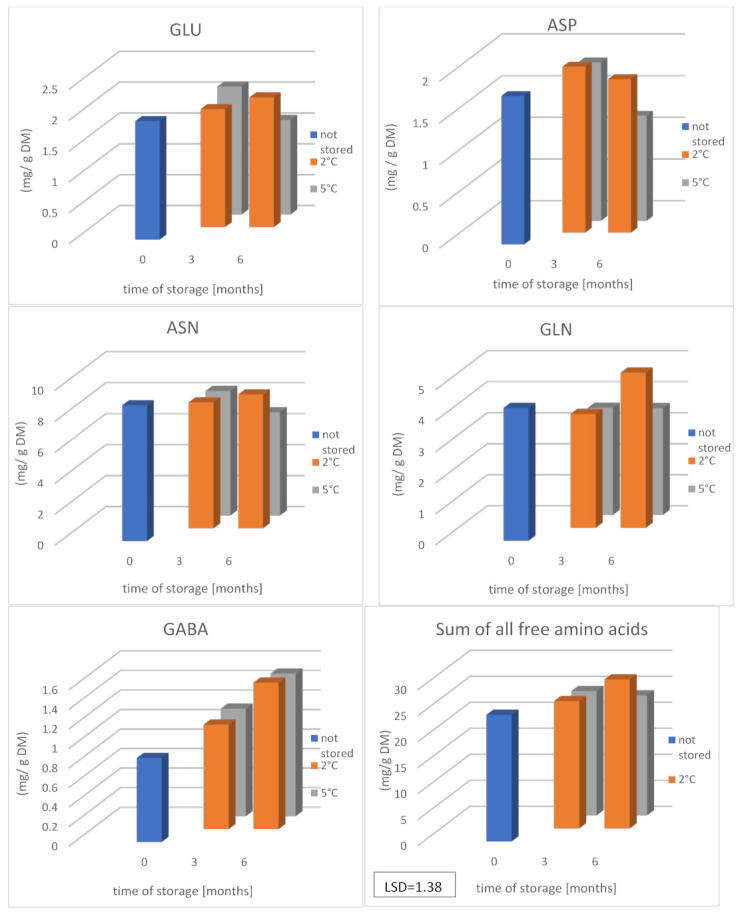
The effect of the temperature and storage time on the content of chosen free amino acids (Asp, Asn, Glu, Gln, and GABA) and the sum of all free amino acids in potatoes of six varieties (average results of six cultivars). Each data value is presented as the mean ± standard deviation (*n* = 12); H26—Herbie 26, RE—Rote Emma, BC—Blue Congo, BA—Blaue Annelise, V—Vineta, and F—Fresco; and Asn—asparagine, Asp—aspartic acid, Gln—glutamine, Glu—glutamic acid, and GABA-γ—aminobutyric acid.

The conducted studies showed that the changes in the content of the sum of all free amino acids and glutamine were clearly increasing in the period of 3–6 months of storage at lower temperatures, i.e., stored (Figure 3), as well as proline (by a maximum of 210%), alanine (by 282%) and serine (by 105%). Table S3 concerned mostly tubers stored at lower than higher (5 °C) temperature. Extending the storage time of the tested potatoes from 3 to 6 months resulted in a significant reduction in the content of aspartic and glutamic acid and asparagine (Figure 3), but not after storage at higher temperatures.

## 3. Materials and Methods

### 3.1. Raw Material

Six potato cultivars (*Solanum tuberosum* L.) were intended for research, including four varieties with coloured flesh and two with a traditional yellow flesh, which were grown in 2012. Tests of approximately 20 kg of potato tubers of the Herbie 26—H26 and Rote Emma—RE varieties with red-fleshed and purple-fleshed Blaue Annelise—BA and Blue Congo—BC potatoes were delivered by the Czech University of Life Sciences in Prague. These potatoes were grown in the experimental plots belonging to testing station of the Central Institute for Supervising and Testing in Agriculture at Přerov nad Labem. Herbie 26 and Blue Congo potatoes came from the Czech Republic Gene Bank, and the Rote Emma and Blaue Annelise varieties from German breeding. Potatoes of yellow flesh varieties collected for the research: Dutch variety Fresco and German variety Vineta were grown in the experimental plots of the Agricultural station of the Wrocław University of Environmental and Life Sciences in Pawłowice near Wrocław in Poland, in Lower Silesia. Přerov nad Labem has a moderately warm oceanic climate and significant rainfall throughout the year, while the vicinity of Wrocław is dominated by a transitional climate that is subject to ocean and continental influences, which means that there is less rainfall. Potatoes used in the experiment differed in their maturity and properties after cooking. Early to medium-early Herbie 26 and Rote Emma cultivars are of fairly firm to floury and of a fairly firm cooking type, respectively. “Blue Congo” belong to medium-early to medium-late maturity and characterize a floury or very floury cooking type, and medium-early Blaue Annelise are of a firm cooking type [4]. Among the yellowish cultivars, very early “Fresco” potatoes characterize a fairly firm cooking type. They are important raw material for french fries, chips, and dried potato processing. On the other side, Vineta there is a very popular early cultivar of consumption potatoes, with high culinary quality, good taste, a firm to fairly firm type of cooking, and a resistance to drought stress and storage conditions [32].

The raw material storage conditions were presented in our previous article [23]. Each sample of 10–15 tubers (weighing approximately 1.5 kg) was stored concurrently in paper bags for zero (at start of storage), 3 and 6 months at two low temperatures (2 °C and 5 °C), and exposed to the air at a constant relative humidity (85% ± 2%; thermohydrometer TH-130 Hama, Mannheim, Germany). After each storage period, the potatoes were analysed. Unpeeled tubers were sliced and then frozen at −18 °C. Frozen samples were placed in a freeze dryer for 24 h. After drying, the samples were ground in a coffee grinder and the obtained freeze-dried potato flour samples were stored at −18 °C in closed polyethylene tubs until further analysis.

### 3.2. Proximate Analysis

The dry-matter content was evaluated according to the Association of Analytical Chemists’ method [33].

### 3.3. Determination of Free Amino Acids

The content of free amino acids was determined using an automatic amino acid analyser (AAA-400, INGOS, Prague, Czech Republic) equipped with an Ostion LG FA ion-exchange column (200 × 3.7 mm, INGOS). Mobile phase was a lithium buffers system with pH 2.7–4.64. The detection was monitored at two wavelengths, i.e., 440 and 570 nm, after a reaction of eluate with ninhydrin (buffered at pH 5.5). The rate flow of lithium buffers was 0.2 mL/min and that of ninhydrin solution—0.3 mL/min. Column temperature was kept at 40–70 °C and detector at 121 °C. The time of one analysis was 209 min. Calculations were performed with the computer program CHROMULAN (Pikron, Prague, Czech Republic), and the results were expressed in mg/g of a sample converted to the dry-matter content. The asparagine (Asn), aspartic acid (Asp), glutamine (Gln), glutamic acid (Glu), arginine (Arg), valine (Val), γ-aminobutyric acid (GABA), proline (Pro), alanine (Ala), tyrosine (Tyr), α-aminoadipic acid (AAA), lysine (Lys), isoleucine (Ile), leucine (Leu), ethanolamine (EA), serine (Ser), threonine (Thr), histidine (His), methionine (Met), phenyloalanin (Phe), glycine (Gly), cysteine (Cys), and ornithine *(L*-orn) were determined.

### 3.4. Statistical Analyses

All data were statistically analysed using Statistica 10.0 (Statsoft, Inc., Tulsa, OK, USA). Homogeneous groups and least significant difference (LSD) values were denoted using Duncan’s multiple comparison test. The significance level was set at α = 0.05, with unidirectional analysis of variance (ANOVA) for three variables.

## 4. Conclusions

The content of free amino acids depends mainly on the potato cultivar, the storage time, and, to a lesser extent, the temperature. The colour of the potato flesh did not affect the content of analysed compounds. The highest amounts of free amino acids were found in the tubers of the red-fleshed “Rote Emma” and purple-fleshed “Blue Congo”, while the lowest amounts of these compounds were found in purple-fleshed “Blaue Annelise” and yellow-fleshed “Vineta” potatoes. In total, asparagine accounted for the largest share of free amino acids (up to 30–36%). In this respect, tubers of the red-fleshed Herbie 26 cultivar and the yellow-fleshed Fresco cultivar stood out. Moreover, the analysed potatoes differed in particular in terms of the content of glutamine, threonine, arginine, serine, lysine, and phenylalanine, as well as the metabolites GABA and α-AAA. Extending the storage period of tubers up to 6 months, particularly at higher temperature (5 °C), contributed to a significant reduction in the content of aspartic and glutamic acids and asparagine in most of the analysed potatoes. There was observed an increase in the content of free amino acids such as proline, alanine, tyrosine, lysine, leucine, serine, threonine, histidine, and the metabolite GABA. The analysed potatoes, after storage at a lower temperature (2 °C), contained on average more total free amino acids than those stored at 5 °C. However, tubers of such cultivars as Rote Emma, Blue Congo, Vineta, and Fresco accumulated more GABA after storage at 5 °C and additionally Vineta cultivars accumulated more aspartic acid and asparagine. The share of asparagine and glutamine in the total of free amino acids depended more on the potato cultivar than on the tuber storage conditions. The red-fleshed potato cultivars Herbie 26 and Rote Emma were characterized by a higher content of asparagine, glutamine, and aspartic and glutamic acids, i.e., amino acids, which gives the potatoes the taste of egumi (spicy, slightly bitter, and tart).

## Figures and Tables

**Table 1:** The effect of temperature and time of storage and potato cultivar on free forms of amino acids contents.

Amino Acid	ANOVA Test						
	Temperature	Time	Cultivar	Temperature × Time	Temperature × Cultivar	Time × Cultivar	Temperature × Time × Cultivar
Asn	***	***	***	***	***	***	***
Asp	***	***	***	***	***	***	***
Gln	***	***	***	***	***	***	***
Glu	*	*	***	***	***	***	***
Arg	***	*	***	***	***	***	***
Val	NS	**	***	*	***	*	***
Gaba	NS	***	***	***	***	***	***
Pro	***	***	***	***	***	***	***
Ala	***	***	***	***	***	***	***
Tyr	NS	***	***	***	***	***	***
Aaa	*	*	***	***	***	***	***
Lys	**	***	***	***	***	***	***
Ile	NS	NS	***	NS	***	***	***
Leu	**	***	***	***	***	***	***
Ea	NS	NS	NS	NS	NS	**	**
Ser	***	***	***	***	***	***	***
Thr	*	***	***	***	***	***	***
His	**	***	***	***	***	***	***
Met	NS	***	***	***	***	***	***
Phe	*	***	***	***	***	***	***
Gly	*	***	***	***	***	***	***
Cys	NS	***	**	***	**	***	***
Orn	NS	*	***	*	**	***	***
Total free aa	***	**	***	***	***	***	***

NS—not significant at * *p* < 0.05; ** *p* < 0.01; and *** *p* < 0.001, respectively.

**Table 2:** Free amino acids, metabolites (mg/g DM), and dry-matter (g/100 g FM) content in potatoes of different flesh colour cultivars as influenced by cultivar (average results of storage time and temperature).

		Potato Cultivar					
		H26	RE	BC	BA	V	F
Amino acid	Asn	9.79 ± 1.79 ^a^	7.98 ± 1.29 ^c^	8.74 ± 1.30 ^b^	5.49 ± 1.27 ^d^	7.70 ± 1.88 ^c^	9.31 ± 1.10 ^a^
	Asp	2.21 ± 0.54 ^a^	1.97 ± 0.52 ^b^	1.39 ± 0.26 ^d^	1.92 ± 0.49 ^b^	1.39 ± 0.36 ^d^	1.66 ± 0.18 ^c^
	Gln	4.62 ± 1.04 ^c^	6.78 ± 1.36 ^a^	5.00 ± 0.96 ^b^	2.14 ± 0.62 ^e^	2.40 ± 0.76 ^e^	3.18 ± 0.96 ^d^
	Glu	1.65 ± 0.34 ^c^	2.04 ± 0.40 ^b^	2.36 ± 0.26 ^a^	2.10 ± 0.33 ^b^	1.66 ± 0.54 ^c^	1.62 ± 0.20 ^c^
	Arg	1.17 ± 0.22 ^c^	1.87 ± 0.32 ^b^	1.98 ± 0.18 ^a^	0.49 ± 0.11 ^f^	0.710.14 ^e^	0.95 ± 0.16 ^d^
	Val	0.91 ± 0.21 ^b^	0.96 ± 0.14 ^b^	1.28 ± 0.16 ^a^	0.66 ± 0.08 ^c^	0.67 ± 0.20 ^c^	1.21 ± 0.20 ^a^
	GABA	0.89 ± 0.23 ^e^	1.26 ± 0.43 ^c^	1.46 ± 0.35 ^b^	1.01 ± 0.40 ^d^	0.57 ± 0.10 ^f^	1.66 ± 0.40 ^a^
	Pro	0.68 ± 0.43 ^c^	0.64 ± 0.28 ^d^	0.88 ± 0.27 ^b^	0.89 ± 0.86 ^b^	0.53 ± 0.10 ^e^	0.98 ± 0.35 ^b^
	Ala	0.22 ± 0.13 ^d^	0.41 ± 0.34 ^b^	0.45 ± 0.24 ^a^	0.35 ± 0.14 ^c^	0.23 ± 0.21 ^d^	0.42 ± 0.17 ^a^
	Tyr	0.43 ± 0.10 ^d^	0.42 ± 0.13 ^d^	0.77 ± 0.06 ^a^	0.52 ± 0.08 ^b^	0.47 ± 0.10 ^c^	0.55 ± 0.14 ^b^
	AAA	0.52 ± 0.69 ^b^	0.20 ± 0.30 ^c^	0.39 ± 0.43 ^b,c^	0.22 ± 0.15 ^c^	0.58 ± 0.58 ^b^	0.86 ± 0.75 ^a^
	Lys	0.62 ± 0.13 ^c^	0.61 ± 0.14 ^c^	0.72 ± 0.11 ^a^	0.31 ± 0.08 ^e^	0.53 ± 0.10 ^d^	0.68 ± 0.16 ^b^
	Ile	0.51 ± 0.11 ^b^	0.52 ± 0.10 ^b^	0.63 ± 0.08 ^a^	0.47 ± 0.06 ^c^	0.31 ± 0.07 ^d^	0.50 ± 0.08 ^b,c^
	Leu	0.24 ± 0.08 ^c^	0.37 ± 0.10 ^a^	0.29 ± 0.02 ^b^	0.25 ± 0.07 ^c^	0.15 ± 0.04 ^d^	0.26 ± 0.08 ^c^
	EA	0.26 ± 0.07 ^a^	0.25 ± 0.06 ^a^	0.34 ± 0.03 ^a^	0.30 ± 0,08 ^a^	0.27 ± 0.05 ^a^	0.29 ± 0.10 ^a^
	Ser	0.71 ± 0.30 ^b^	0.56 ± 0.21 ^c^	0.38 ± 0.05 ^d^	0.39 ± 0.14 ^d^	0.23 ± 0.04 ^e^	0.80 ± 0.30 ^a^
	Thr	0.39 ± 0.09 ^c^	0.47 ± 0.10 ^b^	0.25 ± 0.03 ^e^	0.10 ± 0.02 ^f^	0.36 ± 0.07 ^d^	0.56 ± 0.14 ^a^
	His	0.35 ± 0.09 ^b^	0.35 ± 0,06 ^b^	0.44 ± 0.05 ^a^	0.26 ± 0.04 ^c^	0.28 ± 0.04 ^c^	0.32 ± 0.09 ^b^
	Met	0.30 ± 0.06 ^b^	0.24 ± 0.09 ^c^	0.23 ± 0.04 ^c^	0.15 ± 0.04 ^d^	0.25 ± 0.02 ^c^	0.40 ± 0.06 ^a^
	Phe	0.18 ± 0.06 ^d^	0.21 ± 0.07 ^d^	0.29 ± 0.08 ^c^	0.26 ± 0.05 ^c^	0.35 ± 0.08 ^b^	0.39 ± 0.08 ^b^
	Gly	0.05 ± 0.02 ^d^	0.07 ± 0.02 ^b^	0.06 ± 0.01 ^c^	0.03 ± 0.02 ^f^	0.04 ± 0.01 ^e^	0.09 ± 0.01 ^a^
	Cys	0.07 ± 0.03 ^b^	0.08 ± 0.04 ^a,b^	0.08 ± 0.03 ^a,b^	0.09 ± 0.04 ^a^	0.06 ± 0.03 ^c^	0.09 ± 0.01 ^a^
	*L*-orn	0.05 ± 0.02 ^b^	0.10 ± 0.02 ^a^	0.09 ± 0.03 ^a^	0.09 ± 0.04 ^a^	0.03 ± 0.01 ^b^	0.04 ± 0.02 ^b^
Total free aa (TAA)		26.85 ± 5.44 ^b^	28.36 ± 3.69 ^a^	28.55 ± 2.50 ^a^	18.53 ± 3.31 ^c^	19.81 ± 3.50 ^c^	26.84 ± 4.11 ^b^
Dry matter		23.03 ± 1.03 ^a^	22.58 ± 2.43 ^a,b^	23.30 ± 1.37 ^a^	22.70 ± 1.67 ^a,b^	21.15 ± 1.09 ^c^	20.24 ± 2.85 ^d^

Each data value is presented as the mean ± standard deviation (*n* = 6); ^a–f^ the same letters within the same row are not significantly different; and H26—Herbie 26, RE—Rote Emma, BC—Blue Congo, BA—Blaue Annelise, V—Vineta, and F—Fresco.

**Table 3:** Free amino acids, metabolites (mg/g DM), and dry-matter (g/100 g FM) content in potatoes of different flesh colour cultivars as influenced by temperature of storage.

		Storage Temperature (°C)	
		2	5
Amino acid	Asn	8.51 ± 1.92 ^a^	7.82 ± 2.03 ^b^
	Asp	1.87 ± 0.54 ^a^	1.65 ± 0.44 ^b^
	Gln	4.32 ± 1.90 ^a^	3.72 ± 1.85 ^b^
	Glu	1.98 ± 0.43 ^a^	1.84 ± 0.46 ^b^
	Arg	1.24 ± 0.60 ^a^	1.15 ± 0.58 ^b^
	Val	0.95 ± 0.28 ^a^	0.95 ± 0.30 ^a^
	GABA	1.14 ± 0.45 ^a^	1.14 ± 0.53 ^a^
	Pro	0.87 ± 0.59 ^a^	0.66 ± 0.25 ^b^
	Ala	0.36 ± 0.12 ^a^	0.34 ± 0.19 ^b^
	Tyr	0.53 ± 0.16 ^a^	0.53 ± 0.16 ^a^
	AAA	0.43 ± 0.61 ^a^	0.50 ± 0.51 ^a^
	Lys	0.59 ± 0.19 ^a^	0.56 ± 0.17 ^b^
	Ile	0.49 ± 0.12 ^a^	0.49 ± 0.13 ^a^
	Leu	0.27 ± 0.09 ^a^	0.25 ± 0.10 ^b^
	EA	0.28 ± 0.09 ^a^	0.29 ± 0.10 ^a^
	Ser	0.54 ± 0.31 ^a^	0.48 ± 0.25 ^b^
	Thr	0.35 ± 0.16 ^b^	0.36 ± 0.18 ^a^
	His	0.35 ± 0.10 ^a^	0.32 ± 0.08 ^b^
	Met	0.26 ± 0.09 ^a^	0.27 ± 0.08 ^a^
	Phe	0.29 ± 0.09 ^a^	0.27 ± 0.11 ^b^
	Gly	0.08 ± 0.02 ^a^	0.07 ± 0.02 ^a^
	Cys	0.06 ± 0.03 ^a^	0.06 ± 0.03 ^a^
	*L*-orn	0.07 ± 0.05 ^a^	0.06 ± 0.05 ^a^
Total free aa (TAA)		25.86 ± 5.62 ^a^	23.79 ± 5.22 ^b^
Dry matter		21.30 ± 2.27 ^b^	21.92 ± 2.12 ^a^

Values are means of all cultivars and storage time ± standard deviation; *n* = 36; ^a–b^ the same letters within the same row are not significantly different.

**Table 4:** Free amino acids, metabolites (mg/g DM), and dry-matter (g/100 g FM) content in potatoes of different flesh colour cultivars as influenced by the time of storage.

		Storage Time (Month)		
		0	3	6
Amino acid	Asn	8.77 ± 1.52 ^a^	8.09 ± 1.78 ^b^	7,65 ± 2.47 ^c^
	Asp	1.78 ± 0.42 ^b^	1.94 ± 0.43 ^a^	1.55 ± 0.58 ^c^
	Gln	4.28 ± 1.82 ^a^	3.56 ± 1.66 ^b^	4.22 ± 2.15 ^a^
	Glu	1.92 ± 0.44 ^a,b^	1.99 ± 0.47 ^a^	1.82 ± 0.42 ^b^
	Arg	1.20 ± 0.69 ^a,b^	1.16 ± 0.53 ^b^	1.22 ± 0.57 ^a^
	Val	0.92 ± 0.23 ^b^	0.92 ± 0.22 ^b^	1,00 ± 0.39 ^a^
	GABA	0.86 ± 0.28 ^c^	1.08 ± 0.36 ^b^	1.48 ± 0.56 ^a^
	Pro	0.48 ± 0.18 ^c^	0.66 ± 0.11 ^b^	1.16 ± 0.59 ^a^
	Ala	0.17 ± 0.07 ^c^	0.30 ± 0.12 ^b^	0.57 ± 0.23 ^a^
	Tyr	0.46 ± 0.16 ^c^	0.53 ± 0.14 ^b^	0.59 ± 0.15 ^a^
	AAA	0.34 ± 0.38 ^b^	0.50 ± 0.50 ^a,b^	0.56 ± 0.73 ^a^
	Lys	0.49 ± 0.12 ^c^	0.59 ± 0.17 ^b^	0.66 ± 0.20 ^a^
	Ile	0.49 ± 0.13 ^a^	0.50 ± 0.13 ^a^	0.48 ± 0.12 ^a^
	Leu	0.20 ± 0.06 ^c^	0.25 ± 0.07 ^b^	0.33 ± 0.10 ^a^
	EA	0.29 ± 0.14 ^a^	0.26 ± 0.07 ^a^	0.31 ± 0.07 ^a^
	Ser	0.36 ± 0.12 ^c^	0.48 ± 0.17 ^b^	0,69 ± 0.38 ^a^
	Thr	0.32 ± 0.13 ^c^	0.34 ± 0.15 ^b^	0,40 ± 0.22 ^a^
	His	0.29 ± 0.06 ^c^	0.33 ± 0.08 ^b^	0.38 ± 0.10 ^a^
	Met	0.29 ± 0.09 ^a^	0.25 ± 0.07 ^b^	0.25 ± 0.10 ^b^
	Phe	0.28 ± 0.11 ^b^	0.26 ± 0.09 ^b^	0.30 ± 0.11 ^a^
	Gly	0.05 ± 0.02 ^b^	0.09 ± 0.02 ^a^	0.09 ± 0.03 ^a^
	Cys	0.05 ± 0.03 ^b^	0.06 ± 0.02 ^a^	0.06 ± 0.02 ^a^
	*L*-orn	0.08 ± 0.07 ^a^	0.06 ± 0.03 ^b^	0.06 ± 0.03 ^b^
Total free aa (TAA)		24.38 ± 4.53 ^a^	24.21 ± 4.62 ^a^	25.87 ± 7.02 ^b^
Dry matter		21.04 ± 1.98 ^b^	21.90 ± 2.00 ^a^	21.89 ± 1.52 ^a^

Values are means of all cultivars and storage temperature ± standard deviation; *n* = 24, ^a–b^ the same letters within the same row are not significantly different.

## Data Availability

Data is contained within the article and supplementary materials.

## Supplementary Materials

The following are available online, Table S1: Free amino acids, metabolites (mg/g DM) content in potatoes of different flesh colour varieties as influenced by interaction of variety and time of storage, Table S2: Free amino acids, metabolites (mg/g DM) content in potatoes of different flesh colour varieties as influenced by interaction of variety and temperature of storage, Table S3: Free amino acids, metabolites (mg/g DM) content in potatoes of different flesh colour varieties as influenced by interaction of the storage time and temperature.

## Sample Availability

Samples of the compounds are available from the authors.
