# Supplementary material for: The Free-Amino-Acid Content in Six Potatoes Cultivars through Storage
Source: Molecules. 2021 Mar 2;26(5):1322. doi: 10.3390/molecules26051322 (PMC7958128; doi:10.3390/molecules26051322)
Supplement: Supplementary file 1 [file molecules-26-01322-s001.pdf]

molecules

MDPI

Supplementary Material

The Free-Amino-Acid Content in Six Potatoes Cultivars Through Storage

Anna Pęksa, Joanna Miedzianka *, Agnieszka Nemś and Elżbieta Rytel

Department of Food Storage and Technology, Wroclaw University of Environmental and Life Sciences, Chelmonskiego 37 Street, 51-630 Wroclaw, Poland; anna.peksa@upwr.edu.pl (A.P.); agnieszka.nems@upwr.edu.pl (A.N.); elzbieta.rytel@upwr.edu.pl (E.R.)

* Correspondence: joanna.miedzianka@upwr.edu.pl

Table S1. Free amino acids, metabolites (mg/g DM) content in potatoes of different flesh colour varieties as influenced by interaction of variety and time of storage.

		Potato Cultivar																	
		Herbie 26			Rote Emma			Blue Congo			Blaue Annelise			Vineta			Fresco		
		Time of Storage [months]																	
		0	3	6	0	3	6	0	3	6	0	3	6	0	3	6	0	3	6
Amino acid	Asn	10.21	8.93	10.23	8.74	7.64	7.54	10.41	8.27	7.56	6.54	5.52	4.42	8.47	8.86	5.56	8.26	9.28	10.38
	Asp	2.06	2.33	2.25	2.12	2.45	1.34	1.57	1.55	1.06	2.23	1.88	1.65	1.15	1.83	1.19	1.54	1.62	1.81
	Gln	4.88	3.71	5.27	7.36	6.16	6.83	5.28	4.72	5.00	2.29	1.99	2.15	2.91	2.30	1.98	2.94	2.49	4.10
	Glu	1.66	1.59	1.70	2.07	2.04	2.01	2.58	2.72	2.23	2.19	2.18	1.91	1.48	2.15	1.36	1.51	1.68	1.68
	Arg	1.15	1.05	1.32	2.19	1.54	1.88	2.00	2.03	1.90	0.40	0.56	0.51	0.70	0.81	0.63	0.79	0.97	1.09
	Val*	0.87	0.81	1.06	0.82	0.97	1.08	1.25	1.24	1.33	0.64	0.70	0.65	0.79	0.77	0.45	1.15	1.03	1.46
	GABA	0.77	0.81	1.08	0.88	1.08	1.82	1.13	1.35	1.90	0.56	1.06	1.40	0.52	0.60	0.59	1.27	1.61	2.12
	Pro	0.32	0.58	1.14	0.36	0.63	0.94	0.60	0.82	1.20	0.29	0.61	1.76	0.47	0.60	0.52	0.80	0.72	1.42
	Ala	0.11	0.19	0.38	0.14	0.30	0.80	0.25	0.33	0.76	0.17	0.42	0.47	0.07	0.14	0.48	0.28	0.41	0.56
	Tyr*	0.44	0.37	0.49	0.26	0.46	0.52	0.74	0.72	0.79	0.44	0.57	0.55	0.47	0.50	0.45	0.41	0.50	0.73
	AAA	0.10	0.69	0.76	0.16	0.41	0.05	0.20	0.35	0.62	0.25	0.16	0.27	0.17	0.95	0.62	1.13	0.40	1.04
	Lys*	0.57	0.55	0.74	0.46	0.62	0.74	0.59	0.80	0.77	0.26	0.30	0.37	0.52	0.61	0.47	0.54	0.62	0.88
	Ile*	0.55	0.46	0.53	0.44	0.55	0.58	0.68	0.66	0.55	0.47	0.53	0.42	0.31	0.36	0.26	0.46	0.47	0.57
	Leu*	0.20	0.22	0.31	0.26	0.35	0.49	0.29	0.28	0.30	0.18	0.27	0.30	0.10	0.16	0.18	0.19	0.23	0.36
	EA	0.29	0.20	0.28	0.22	0.23	0.30	0.32	0.33	0.36	0.43	0.23	0.25	0.28	0.27	0.27	0.20	0.27	0.41
	Ser	0.50	0.59	1.05	0.33	0.58	0.76	0.35	0.37	0.41	0.26	0.41	0.49	0.21	0.25	0.22	0.53	0.68	1.20
	Thr*	0.41	0.35	0.41	0.36	0.45	0.58	0.26	0.27	0.25	0.10	0.10	0.11	0.34	0.41	0.33	0.46	0.48	0.73
	His	0.30	0.32	0.44	0.28	0.34	0.42	0.40	0.46	0.48	0.26	0.25	0.26	0.27	0.31	0.26	0.24	0.29	0.44
	Met*	0.30	0.28	0.31	0.24	0.24	0.25	0.27	0.22	0.20	0.17	0.15	0.14	0.30	0.25	0.19	0.43	0.34	0.44
	Phe*	0.19	0.14	0.21	0.14	0.20	0.28	0.37	0.25	0.26	0.24	0.27	0.28	0.37	0.40	0.29	0.36	0.32	0.50
	Gly	0.03	0.05	0.06	0.06	0.07	0.09	0.06	0.07	0.07	0.02	0.05	0.04	0.05	0.05	0.03	0.08	0.09	0.10
	Cys*	0.05	0.07	0.08	0.03	0.10	0.10	0.03	0.10	0.10	0.06	0.12	0.10	0.02	0.09	0.08	0.09	0.08	0.08
	L-orn	0.04	0.05	0.07	0.12	0.08	0.10	0.09	0.11	0.07	0.19	0.04	0.04	0.03	0.03	0.02	0.03	0.06	0.05
	Total free aa (TAA)	26.03	24.32	30.20	28.06	27.51	29.51	29.82	27.67	28.17	18.67	18.37	18.54	20.03	22.73	16.66	23.71	24.66	31.15

*essential amino acid

Table S2. Free amino acids, metabolites (mg/g DM) content in potatoes of different flesh colour varieties as influenced by interaction of variety and temperature of storage.

		**Potato Cultivar**											
		**Herbie 26**		**Rote Emma**		**Blue Congo**		**Blaue Annelise**		**Vineta**		**Fresco**	
		**Temperature of Storage [°C]**											
		**2**	**5**	**2**	**5**	**2**	**5**	**2**	**5**	**2**	**5**	**2**	**5**
Amino acid	Asn	11.43	7.73	8.22	6.97	8.15	7.68	5.61	4.32	6.76	7.86	10.13	9.54
	Asp	2.91	1.67	1.97	1.81	1.29	1.32	2.04	1.49	1.44	1.59	1.83	1.60
	Gln	5.22	3.76	7.06	5.59	5.47	4.25	2.64	1.49	2.65	1.63	2.96	3.63
	Glu	2.03	1.27	2.08	1.97	2.30	2.20	2.10	1.99	1.87	1.65	1.66	1.70
	Arg	1.40	0.97	1.72	1.69	2.05	1.88	0.61	0.46	0.63	0.80	1.13	0.92
	Val*	1.12	0.74	0.99	1.06	1.20	1.37	0.73	0.61	0.50	0.72	1.24	1.24
	GABA	1.15	0.74	1.40	1.49	1.54	1.70	1.38	1.08	0.54	0.64	1.69	2.03
	Pro	1.10	0.62	0.83	0.74	1.09	0.93	1.68	0.69	0.61	0.51	1.13	1.00
	Ala	0.26	0.30	0.68	0.41	0.58	0.51	0.43	0.46	0.39	0.24	0.36	0.61
	Tyr*	0.52	0.33	0.47	0.51	0.81	0.74	0.55	0.57	0.40	0.55	0.59	0.64
	AAA	0.67	0.79	0.34	0.11	0.28	0.70	0.08	0.34	0.39	1.19	1.08	0.36
	Lys*	0.74	0.54	0.66	0.70	0.83	0.75	0.38	0.29	0.46	0.62	0.79	0.72
	Ile*	0.59	0.39	0.51	0.62	0.59	0.62	0.47	0.47	0.29	0.32	0.54	0.50
	Leu*	0.33	0.20	0.39	0.45	0.29	0.29	0.32	0.24	0.15	0.19	0.30	0.30
	EA	0.27	0.21	0.22	0.32	0.34	0.35	0.24	0.24	0.23	0.30	0.33	0.35
	Ser	1.00	0.64	0.75	0.60	0.41	0.36	0.49	0.42	0.20	0.27	0.90	0.98
	Thr*	0.47	0.29	0.51	0.53	0.23	0.29	0.12	0.09	0.31	0.43	0.54	0.67
	His	0.46	0.30	0.38	0.39	0.49	0.45	0.29	0.23	0.26	0.31	0.39	0.33
	Met*	0.33	0.26	0.25	0.24	0.21	0.20	0.15	0.14	0.20	0.24	0.42	0.36
	Phe*	0.23	0.12	0.24	0.24	0.31	0.20	0.30	0.25	0.31	0.37	0.42	0.40
	Gly	0.07	0.04	0.09	0.07	0.07	0..07	0.05	0.03	0.03	0.04	0.09	0.10
	Cys*	0.09	0.06	0.12	0.09	0.10	0.10	0.11	0.11	0.08	0.08	0.08	0.09
	L-orn	0.07	0.05	0.09	0.09	0.10	0.08	0.06	0.03	0.03	0.02	0.07	0.04
	Total free aa (TAA)	32.46	22.04	29.98	27.04	28.77	27.07	20.85	16.07	18.80	20.59	28.68	28.13

Table S3. Free amino acids, metabolites (mg/g DM) content in potatoes of different flesh colour varieties as influenced by interaction of the storage time and temperature.

		Time of Storage [months]				
		0	3		6	
			Temperature of Storage [°C]			
			2	5	2	5
Amino acid	Asn	8.77	8.13	8.04	8.42	8.66
	Asp	1.78	1.99	1.90	1.82	1.26
	Gln	4.28	3.67	3.45	5.22	3.44
	Glu	1.92	1.91	2.07	2.13	1.53
	Arg	1.20	1.22	1.09	1.30	1.15
	Val*	0.92	0.92	0.92	0.97	1.00
	GABA	0.86	1.07	1.10	1.47	1.46
	Pro	0.47	0.68	0.64	1.46	0.86
	Ala	0.17	0.27	0.33	0.65	0.52
	Tyr*	0.46	0.52	0.54	0.59	0.58
	AAA	0.33	0.57	0.42	0.18	0.74
	Lys*	0.49	0.60	0.57	0.67	0.64
	Ile*	0.48	0.51	0.50	0.48	0.48
	Leu*	0.20	0.26	0.24	0.34	0.31
	EA	0.29	0.26	0.25	0.28	0.34
	Ser	0.36	0.47	0.49	0.74	0.60
	Thr*	0.32	0.34	0.34	0.36	0.42
	His	0.29	0.35	0.31	0.40	0.36
	Met*	0.29	0.27	0.23	0.23	0.27
	Phe*	0.28	0.26	0.26	0.32	0.27
	Gly	0.05	0.06	0.06	0.07	0.06
	Cys*	0.05	0.09	0.10	0.11	0.08
	L-orn	0.08	0.07	0.05	0.07	0.05
	Total free aa (TAA)	24.38	24.51	23.91	28.31	23.07
